# AST-to-ALT ratio in the first trimester and the risk of gestational diabetes mellitus

**DOI:** 10.3389/fendo.2022.1017448

**Published:** 2022-09-29

**Authors:** Rongjing An, Shujuan Ma, Na Zhang, Huijun Lin, Tianyu Xiang, Mengshi Chen, Hongzhuan Tan

**Affiliations:** ^1^ Hunan Provincial Key Laboratory of Clinical Epidemiology, Xiangya School of Public Health, Central South University, Changsha, China; ^2^ Department of Epidemiology and Health Statistics, Xiangya School of Public Health, Central South University, Changsha, China; ^3^ Reproductive and Genetic Hospital of CITIC‑Xiangya, Clinical Research Center for Reproduction and Genetics in Hunan Province, Changsha, China

**Keywords:** gestational diabetes mellitus, alanine transaminase, aspartate aminotransferase, nomogram, predictive value

## Abstract

**Background:**

Aspartate aminotransferase-to-alanine transaminase ratio (AST/ALT) has been reported affect the risk of type 2 diabetes (T2DM), but it is uncertain if it has relationship with gestational diabetes mellitus (GDM).

**Objectives:**

Our study aimed to investigate the association between AST/ALT ratio in the first trimester and the risk of subsequent development of GDM.

**Method:**

This prospective cohort study enrolling 870 pregnant women, 204 pregnant women with missing data or liver diseases were excluded, 666 pregnant women were included in this study containing 94 GDM women. Blood samples were collected in the first trimester. Univariate analysis and multivariate logistic regression were used to evaluate the association between AST/ALT and GDM. Nomogram was established based on the results of multivariate logistic analysis. Receiver Operating Characteristic (ROC) curves and calibration curves were used to evaluate the predictive ability of this nomogram model for GDM. Decision curve analysis (DCA) was used to examine the clinical net benefit of predictive model.

**Results:**

AST/ALT ratio (RR:0.228; 95% CI:0.107-0.488) was associated with lower risk of GDM after adjusting for confounding factors. Indicators used in nomogram including AST/ALT, maternal age, preBMI, waist circumference, glucose, triglycerides, high density lipoprotein cholesterol and parity. The area under the ROC curve (AUC) value of this predictive model was 0.778, 95% CI (0.724, 0.832). Calibration curves for GDM probabilities showed acceptable agreement between nomogram predictions and observations. The DCA curve demonstrated a good positive net benefit in the predictive model.

**Conclusions:**

The early AST/ALT level of pregnant women negatively correlated with the risk of GDM. The nomogram including AST/ALT at early pregnancy shows good predictive ability for the occurrence of GDM.

## Introduction

For Gestational diabetes mellitus (GDM), the major pregnancy-related endocrinopathy, has been steadily increasing worldwide in many countries over recent decades (1). According to the International Diabetes Association in 2021, 51 studies from 41 countries reported that among pregnant women aged 20-49 years, the incidence of hyperglycemia was 16.7%, and 70%-90% of hyperglycemia cases were caused by GDM, 2021 (2). In China, approximately 17.6% of pregnant women suffered from GDM (3). GDM is associated not only with impaired glucose tolerance or type 2 diabetes mellitus (T2DM) after giving birth to women (4) but also has short- and long-term effects on children such as excessive fetal development, preterm delivery, increased incidence of T2DM, and obesity (5). Therefore, early detection of GDM through screening programs is essential to treat and prevent such diseases and to advance appropriate management. Currently, random blood glucose, fasting blood glucose and oral glucose tolerance tests (OGTT) are usually used to predict or identify GDM. However, such testing is expensive, time-consuming, and cannot anticipate or detect all cases (6); therefore, knowledge of new biomarkers for predicting GDM is crucial.

The liver, an organ essential for maintaining glucose homeostasis and insulin resistance, is important in the pathogenesis of metabolic syndrome (7). Liver transaminases such as alanine aminotransferase (ALT) and aspartate aminotransferase (AST) are usually used to assess the health of the liver (8) especially enable to reflect the accumulation of fat in the liver (9). The serum AST/ALT ratio, a feature of viral hepatitis, was first proposed by Fernando De Ritis in 1957 (10). However, the AST/ALT ratio is not only used to assess liver disease but also cardiovascular disease (11), chronic kidney disease (12) and metabolic syndrome (13). A previous study found that AST/ALT can affect the risk of T2DM (14). The AST/ALT ratio independently has negative association with the risk of developing T2DM, and the relationship was non-linear (15). In addition, the measurement of these liver enzymes involves well-standardized, simple, inexpensive, and routine tests that do not require fasting before venipuncture, suggesting that they could be incorporated in scores to predict diabetes risk (16). Since the pathogenesis of GDM is similar to that of T2DM, it is suggested that AST/ALT ratio may correlate with the risk of GDM. To date, only one study has focused on the association between AST/ALT in early pregnancy and subsequent GDM (17), which reported that ALT/AST was independently associated with the incidence of GDM. However, some potential confounding factors were not adjusted in this study, and AST/ALT predictive value in GDM have not valued. Therefore, it is of interest to explore the association between AST/ALT levels and the occurrence of GDM and investigate its predictive value for GDM.

This study aimed to determine whether AST/ALT of pregnant women in the first trimester was associated with the risk of subsequent development of GDM and their potential predictive value for GDM.

## Methods

### Study population and design

The pregnant women in the first trimester were recruited in Hunan Maternal and Child Health Hospital from Mar. 2017 to Dec. 2018. The inclusion criteria were as follows: natural conception; no history of diabetes or GDM, hypertension, thyroid and cardiovascular and cerebrovascular diseases before pregnancy; cases with renal disease or collagen vascular diseases; no acute infection in the last 2 weeks and no antibiotics during pregnancy; not taking drugs that may affect glucose metabolism; planning to complete obstetric examination and delivery in Hunan Maternal and Child Health Hospital; pregnant women who voluntarily participate in the project at 10-13^+6^ weeks of pregnancy. The questionnaire data and blood samples of these participants were collected from the first trimester and followed up until the 2 h, 75-g OGTT (24-28 weeks) was performed to diagnose GDM. According to the purpose of this report, we excluded participants whose ALT, AST as well as the 3-point OGTT results were missing and women with liver diseases. All participants provided written informed consent, and the study was approved by the Medical Ethics Committee of Hunan Maternal and Child Health Hospital (no. EC201624).

### Ascertainment of outcome

The diagnostic criteria for GDM in this study were the 2011 IADPSG criteria (18). GDM was defined at 24–28 gestational weeks based on the results of the OGTT. Pregnant women were diagnosed with GDM when their applied glucose level was elevated in one or more of the following: fasting blood glucose (FBG) ≥ 5.1 mmol/L, 1 hour blood glucose (1-hBG) ≥ 10.0 mmol/L, 2 hours blood glucose (2-hBG) ≥ 8.5 mmol/L.

### Measurement of liver enzymes and other clinical indicators

Blood samples were collected during the first trimester and stored at −80°C. ALT, AST, blood glucose and blood lipids containing triglycerides (TG), total cholesterol (TC), high-density lipoprotein cholesterol (HDL-C), low-density lipoprotein cholesterol (LDL-C) were tested in the first trimester. All assays were performed at the Hunan Provincial Maternal and Child Health Hospital Laboratory. All measurements were performed in duplicate and the results were reported as the mean.

### Questionary data

Some questionnaire data were also collected during the first trimester, which included family history of diabetes, hyperlipidemia, and hypertension (yes, no), alcohol consumption (yes, no), the weight of pregnant women from pre-pregnancy to second trimester and height, maternal age, parity, waist circumference (WC), and exercise for more than 30 minutes before pregnancy (yes, no). Pre-pregnancy body mass index(preBMI) was calculated as pre-pregnancy weight/height^2^ (kg/m^2^). Gestational weight gain was calculated by first and second trimester weights.

### Calculation of sample size

We calculated sample size using PASS 11.0. A meta-analysis in mainland China found that incidence of GDM in older pregnant women was 26.7% (P1 = 26.7%), whereas that in younger pregnant women was just 13.4% (P2 = 13.4%), α=0.05, β=0.1. The risk of developing GDM in those exposed to other suspicious risk factors (such as obesity, family history of diabetes, etc.) concerned in this study is mostly higher than 26.7% (19). According to the calculation, a sample size of at least 378 participants are required to develop the GDM prediction model.

### Statistical analysis

The Kolmogorov–Smirnov equality of distributions test was used to check the normality of continuous variables, median and interquartile range (IQR) describing continuous variables that were not normally distributed, mean ± standard deviation (SD) was used to describe normally distributed continuous variables, and percentages were used to describe categorical variables. Differences between the GDM and non-GDM groups were analyzed using the t-test or Wilcoxon rank-sum test and the Chi-square test or Fisher’s exact test for continuous and categorical variables, respectively. Multivariate logistic regression analysis adjusted for covariates which have significant differences in univariate analysis was performed to assess the association of AST/ALT with subsequent risk of GDM.A prediction nomogram was constructed based on the results of multivariate logistic regression. We plotted the receiver operating characteristic (ROC) curve to evaluate the nomogram, and a calibration curve (Hosmer-Lemeshow test) was used to assess the goodness of fit. To determine the clinical utility of the nomogram, decision curve analysis was applied to GDM patients by quantifying the net benefit at different threshold probabilities. We performed a correlation analysis of the diagnostic results of OGTT and AST/ALT. Statistical significance was set at *P* value < 0.05 (two-tailed). All analyses were conducted using SPSS22.0 and R 4.2.1 with R packages caret, rmda, rms, regplot, and pROC.

## Results

### Basic characteristics of cohort

A total of 870 pregnant women were enrolled and followed up until the outcome, 204 were excluded because ALT, AST, or 3-point OGTT results were incomplete, or because pregnant women had liver diseases. Finally, 666 participants were included in the study, of whom 94 were diagnosed with GDM, with an incidence of 14.1%.

The basic characteristics of the study cohort are shown in **Table 1**. The average age of pregnant women with GDM (31.93 ± 4.69 years) was significantly higher than that of normal women (29.15 ± 3.92 years). GDM cases had higher preBMI, WC, and percentage of parity≥1 than those in non-GDM women.

**Table 1:** Basic Characteristics of GDM and non-GDM pregnant women.

Characteristics	GDM (n = 94)		Non-GDM (n = 572)	*P*
Maternal age	31.93 ± 4.69		29.15 ± 3.92	<0.001
PreBMI(kg/m^2^)	22.00 ± 2.82		20.44 ± 2.50	<0.001
WC (cm)	80.93 ± 8.24		77.86 ± 7.76	<0.001
Gestational weight gain	5.53 ± 3.79		5.44 ± 2.89	0.840
Parity				0.023
≧1	48 (51.1%)		220 (38.5%)	
0	46 (48.9%)		352 (61.5%)	
Family history of hypertension				0.35
yes	28 (29.8%)		200 (35.0%)	
no	66 (70.2%)		372 (65.0%)	
Family history of diabetes				0.099
yes	13 (13.8%)		51 (8.92%)	
no	81 (86.2%)		521 (91.1%)	
Alcohol consumption in first trimester				0.508
yes	4 (4.3%)		16 (2.8%)	
no	90 (95.7%)		556 (97.2%)	
Exercise more than 30 min pre-pregnancy				0.539
yes	20 (21.3%)		162 (28.3%)	
no	74 (78.7%)		410 (71.7%)	

As for the clinical parameters in the first trimester (shown in **Table 2**), GDM women had significantly higher glucose (4.87 vs. 4.64), TG (1.55 vs. 1.33) and ALT (18.00 vs. 14.30) levels, but a lower AST/ALT ratio (0.96 vs. 1.18) and HDL-C level (1.83 vs. 1.98).

**Table 2:** Clinical parameters in GDM and non-GDM pregnant women in the first trimester.

Characteristics	GDM (n = 94)	Non-GDM (n = 572)	*P*
ALT(UI/L)	18.00 (13.10,25.93)	14.30 (10.30,18.60)	<0.001
AST(UI/L)	15.88 (17.90,21.90)	17.70 (14.10,20.10)	0.054
AST/ALT	0.96 (0.79,1.21)	1.18 (1.02,1.49)	<0.001
Glucose(mmol/L)	4.87 ± 0.41	4.64 ± 0.44	<0.001
TG(mmol/L)	1.55 (1.22,1.95)	1.33 (1.75,1.08)	<0.001
LDL-C(mmol/L)	2.59 ± 0.65	2.45 ± 0.67	0.066
TC(mmol/L)	4.63 ± 0.73	4.57 ± 0.79	0.454
HDL-C(mmol/L)	1.83 ± 0.43	1.98 ± 0.41	0.001

### Multivariable analysis and model construction

Multivariate logistic regression analysis was performed to determine whether AST/ALT ratio had association with the risk of GDM (**Table 3**). After adjusting for maternal age, preBMI, glucose, WC, TG, HDL-C, and parity (variables with significant differences in univariate analysis), the results demonstrated that with an increase in AST/ALT, the risk for the development of GDM will decrease (RR:0.228; 95% CI: 0.107-0.488).

**Table 3:** Results of multivariable logistic regression for GDM.

Model	B	RR (95%Confidence Interval)	*P*
AST/ALT	-1.476	0.228 (0.107~0.488)	<0.001
Maternal age	0.112	1.119 (1.038~1.195)	<0.001
PreBMI	0.085	1.088 (0.971~1.220)	0.195
WC	-0.013	0.987 (0.952~1.024)	0.494
Parity	-0.124	0.884 (0.499~1.626)	0.872
Glucose	0.987	2.682 (1.488~4.830)	0.001
TG	0.391	1.478 (0.984~2.219)	0.060
HDL-C	-0.410	0.664 (0.357~1.232)	0.194

### Establishment and evaluation of a predictive nomogram

**Figure 1:**
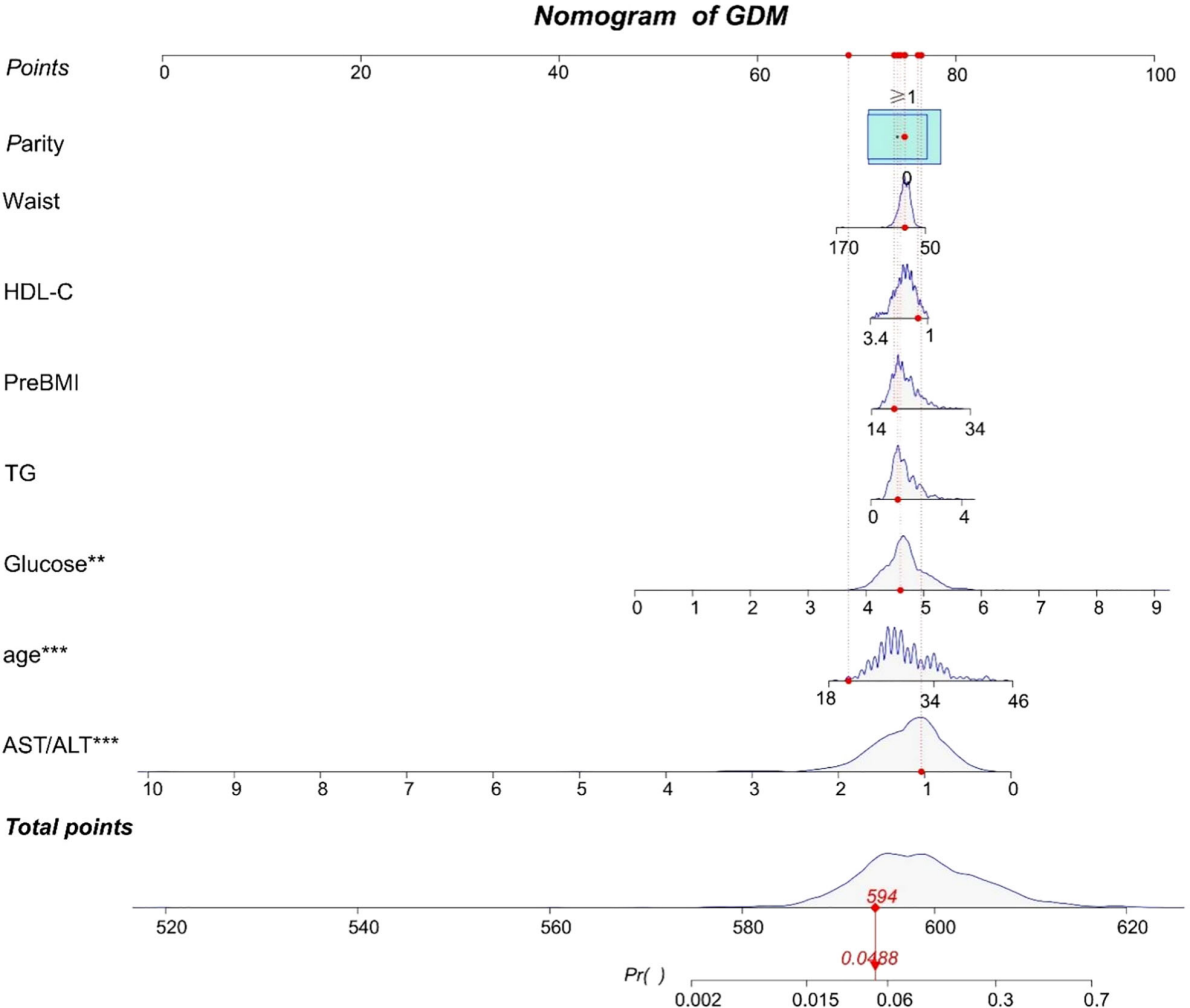
A nomogram to predict for the risk of GDM. Scores are calculated by aligning the dots on each numbered row with the dots of the”Points” row. The total score is obtained by adding up all the scores and plotted on the “Total points” line. The difference in the relative proportion of patients in parity (0, ≥1) is represented by the rectangular area. Participant 1 in our study is listed as an example (expressed in red). Her total score was 594, which indicating that her probability of GDM was 4.88%. **P value < 0.01 and ***P value < 0.0001.

**Figure 2:**
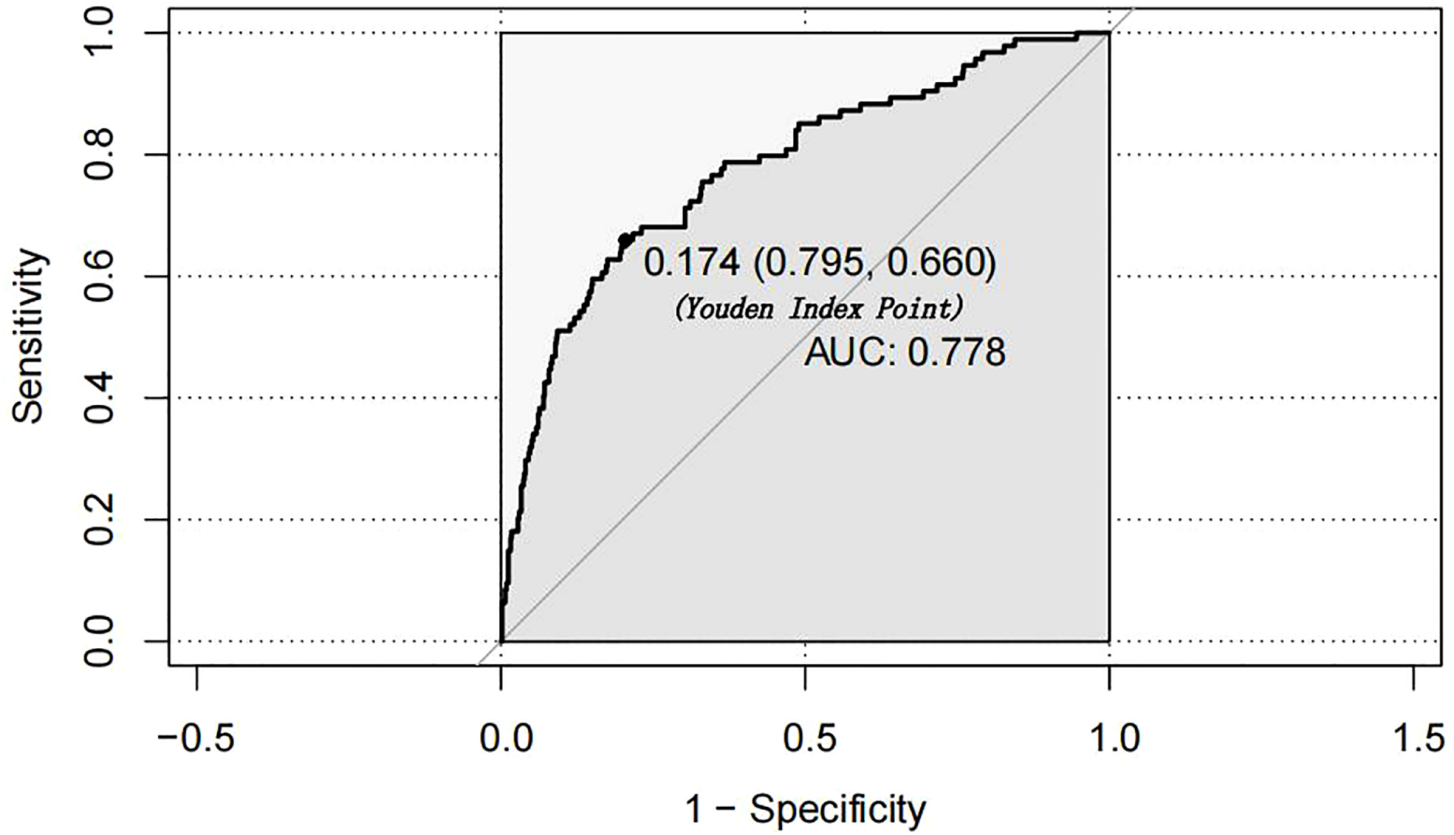
Receiver operating characteristic (ROC) curves of nomogram.

**Figure 3:**
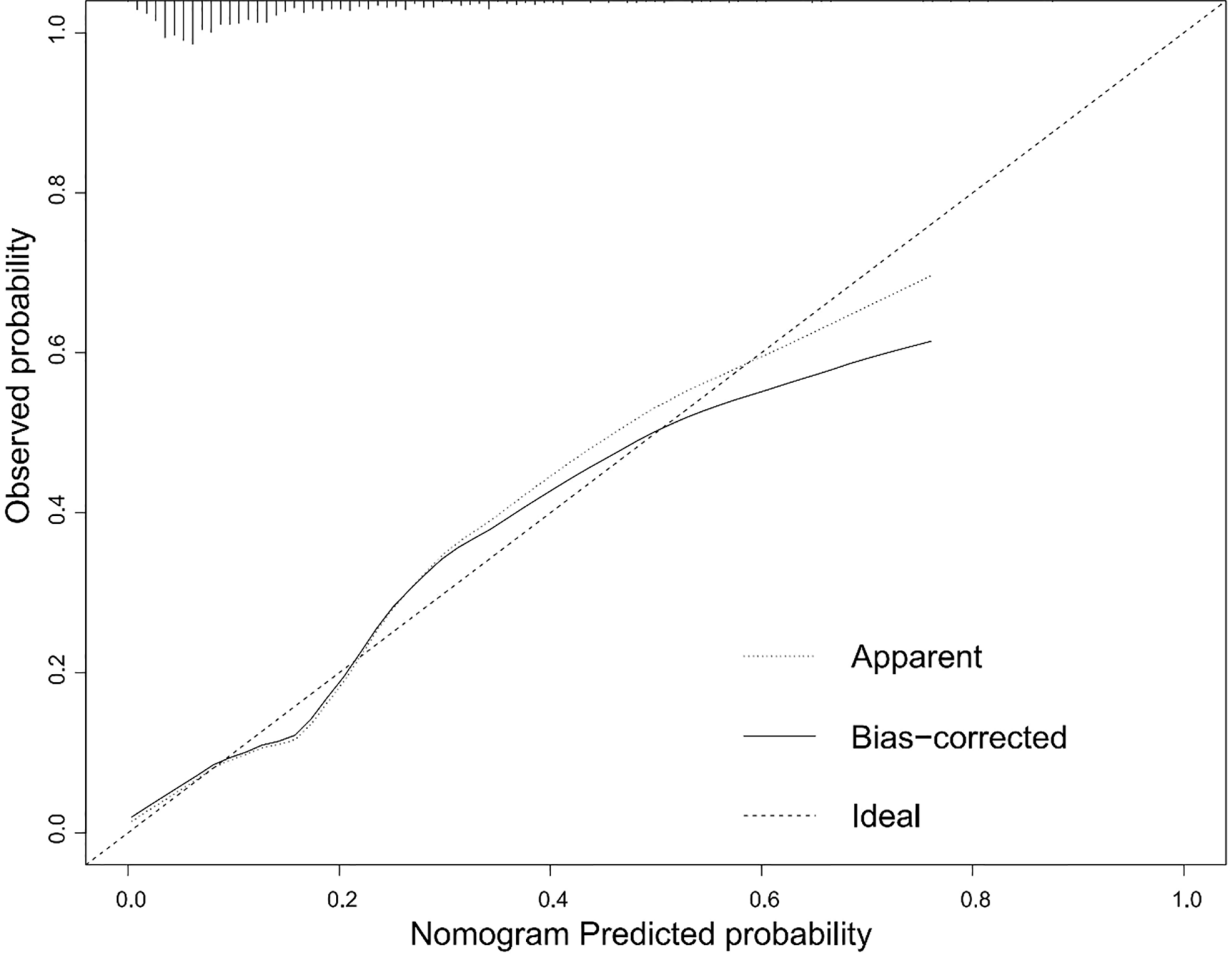
Calibration curves for the nomogram. The x-axis represents the predicted rate of GDM. The y-axis shows observed probability of GDM occurrence. The dashed diagonal line is the ideal line. The line adjacent to the ideal line represents the predictive accuracy.

**Figure 4:**
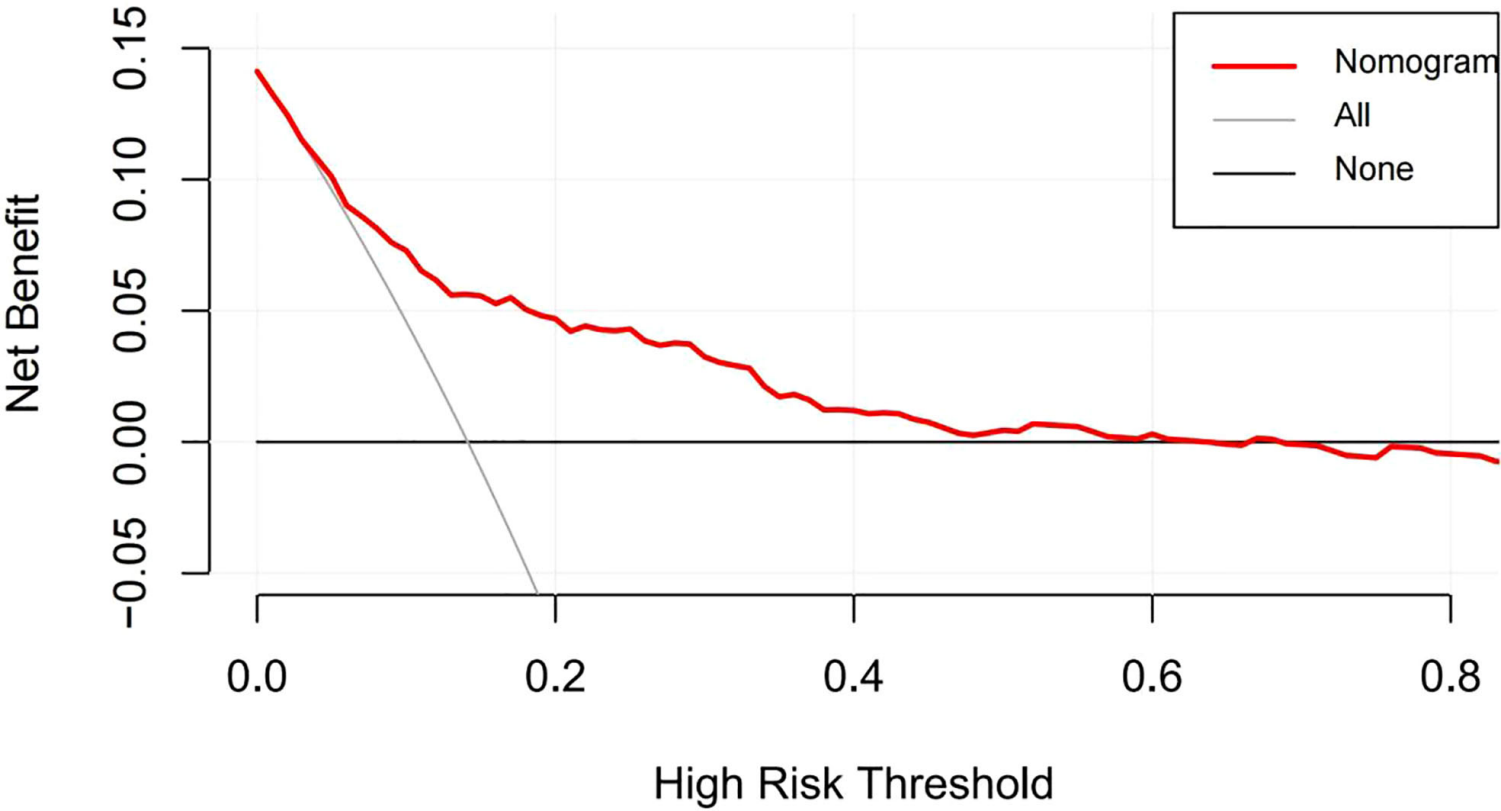
Decision curve analysis of the nomogram. The red line represents the clinical net benefits according to the threshold probabilities; The horizontal line assumes that net benefit when no one develops a GDM; the solid gray line indicates the net benefit that all cases suffer GDM.

According to the results of the logistic regression analyses, a nomogram that could predict the occurrence of GDM was established (**Figure 1**). The area under the ROC curve (AUC) value of this model (**Figure 2**) was 0.778 (95% CI:0.724~0.832, *P*<0.001). Calibration curve shows good agreement between predicted and actual results (**Figure 3**), c-index was 0.778 and the Hosmer-Lemeshow test *p* value was 0.683. AST/ALT was significant correlated with the incidence of GDM(r =-0.177,*p*<0.001). Finally, DCA plot showed that the predictive nomogram model provided good net positive benefit for most threshold probabilities (**Figure 4**).

## Discussion

In this cohort study, we examined the relationship of AST-to -ALT ratio in early pregnancy and the incidence of GDM in Chinese pregnant women. The ratio of AST/ALT increased, and the risk of occurrence to GDM decreased. In addition, a predictive nomogram of GDM that included AST/ALT levels in early pregnancy was established, which showed good discrimination and clinical usability for predicting the development of GDM.

Liver transaminases, AST and ALT, are widely reported that has close relationship with the occurrence of T2DM (20–22). However, studies on the relationship between liver transaminases and GDM are limited and conflicting. Leng J et al. reported that elevated ALT levels in the first trimester, even within normal range, are associated with the risk of GDM (23). Other researchers found that liver transaminases, including ALT and AST, cannot predict GDM (24). AST/ALT as a liver marker has been recently reported is correlated with metabolic diseases. Elevated ALT levels and low AST/ALT ratios have been discovered to be associated with insulin resistance (IR) (25), and AST/ALT was considered as a surrogate marker for IR and hyperinsulinemia (26). Moreover, the AST/ALT ratio was found to be negatively associated with the incidence of T2DM and was demonstrated to be one of the best predictors of metabolic syndrome and T2DM in the Asian population (27–29). However, only one study investigated the relationship between AST/ALT levels in the first trimester and GDM. Consistent with our study, they reported that lower AST/ALT in early pregnancy, even within the normal range, was an independent risk factor for GDM (17). Compared to this study, our study included and adjusted for more confounding variables to make the results more reliable.

Although the potential mechanism underlying the relationship between the AST/ALT ratio and GDM remains unclear, several speculations exist. The liver minimizes postprandial glucose fluctuations by absorbing and storing glucose (30). Liver damage can affect postprandial glucose, since 60-65% of the oral glucose load is processed by the liver (31). The level of ALT and AST can reflect fat accumulation in the liver (32) and decreased AST/ALT is considered a biomarker of nonalcoholic fatty liver diseases (NAFLD), even if its value is within the normal range (33). NAFLD can lead to hepatic IR and is considered a feature of metabolic syndrome (34), which causes more production of glucose by the liver (20), affecting human blood sugar and the occurrence of GDM. On the other hand, another study also reported that a positive correlation existed between ALT and fasting glucagon levels which suggesting an interaction exists between the liver and α-cell function (35), which may affect the blood glucose metabolism and leads to GDM.

In our study, we first constructed a nomogram of GDM, which demonstrated that AST/ALT ratio in the first trimester could be used to predict GDM. The nomogram can be widely used as a personalized risk prediction tool with an intuitive digital interface and improved accuracy, which making it easy for clinicians and pregnant women to calculate the risk of GDM based on their own conditions. Various first-trimester nomograms for GDM have been proposed; however, most clinical indicators in these models include only blood glucose and lipids, and liver transaminases were not included. The predictive performance of these nomograms was criticized as having limited diagnostic accuracy, and the AUC of these models ranged from 0.690-0.770, which was less accurate than that of our model (36–38). Moreover, some prediction models containing a number of new biomarkers, such as high molecular weight adiponectin, omentin-1 (39), putrescine (40), and RNA (41), yet have high AUC values; however the detection technology is complex and expensive making them unsuitable for routine screening prediction. In our model, maternal age, preBMI, parity, WC, blood glucose, TG, HDL-C and AST/ALT could be easily measured in the first 3 months of pregnancy with good practicality. In addition, the results of the DCA curve also proved that our model had a positive effect confirming the clinical value of the model.

There are some strengths and weaknesses to our study. Our study was a prospective cohort study that could better explain the causal relationship between AST/ALT levels in the first trimester and GDM. In addition, we first established a nomogram including AST/ALT to predict GDM and demonstrated that model has better discrimination and predictive value. However, limitations also existed in our study. First, limited by actual survey results, although the prevalence of GDM in the cohort was close to the national level, the cases in our cohort was small, this may result in less statistically significant results for some factors with small associations, while increasing the confidence interval for the results. Second, our study involved a single-center cohort, which is not representative of the entire Chinese GDM population. Further evaluating external validity and updating the nomogram in large, multicenter study populations is imperative. Third, because of the robustness of the data, we did not monitor the dynamic changes in AST/ALT ratio from the first to the second trimester and were unable to explore the impact of these dynamic changes on the risk of GDM. Further studies could focus on this topic.

## Conclusion

In conclusion, the AST/ALT ratio in the first trimester negatively correlated with the risk of GDM. The nomogram for GDM, including AST/ALT at early pregnancy, shows favorable discrimination and predictive value.

## Data availability statement

The raw data supporting the conclusions of this article will be made available by the authors, without undue reservation.

## Ethics statement

The studies involving human participants were reviewed and approved by Medical Ethics Committee of Hunan Maternal and Child Health Hospital. The patients/participants provided their written informed consent to participate in this study.

## Author contributions

RA, HT and MC designed the idea and method of this study. Validation, SM, NZ, HL and TX organize and verify the data of this study. SM, RA and NZ analyzed the data. RA and SM wrote the manuscript. HT and MC supervised and supported the study. All authors contributed to the article and approved the submitted version.

## Funding

This research was funded by National Natural Science Foundation of China (grant # 81773535, 81973137 and 82173608) and Key Research and Development Program of Hunan Province (grant # 2018SK2061).

## Conflict of interest

The authors declare that the research was conducted in the absence of any commercial or financial relationships that could be construed as a potential conflict of interest.

## Publisher’s note

All claims expressed in this article are solely those of the authors and do not necessarily represent those of their affiliated organizations, or those of the publisher, the editors and the reviewers. Any product that may be evaluated in this article, or claim that may be made by its manufacturer, is not guaranteed or endorsed by the publisher.
